# Supplementary material for: Yeast Nup84-Nup133 complex structure details flexibility and reveals conservation of the membrane anchoring ALPS motif
Source: Nat Commun. 2020 Nov 27;11:6060. doi: 10.1038/s41467-020-19885-5 (PMC7695694; doi:10.1038/s41467-020-19885-5)
Supplement: Supplementary file 1 — Supplementary Information [file 41467_2020_19885_MOESM1_ESM.pdf]

Supplementary Information for:

Yeast Nup84-Nup133 complex structure details flexibility and reveals conservation of the membrane anchoring ALPS motif

Sarah A. Nordeen[1], Daniel L. Turman[1] & Thomas U. Schwartz[1*]

[1]Department of Biology, Massachusetts Institute of Technology, Cambridge, Massachusetts, United States
*corresponding author

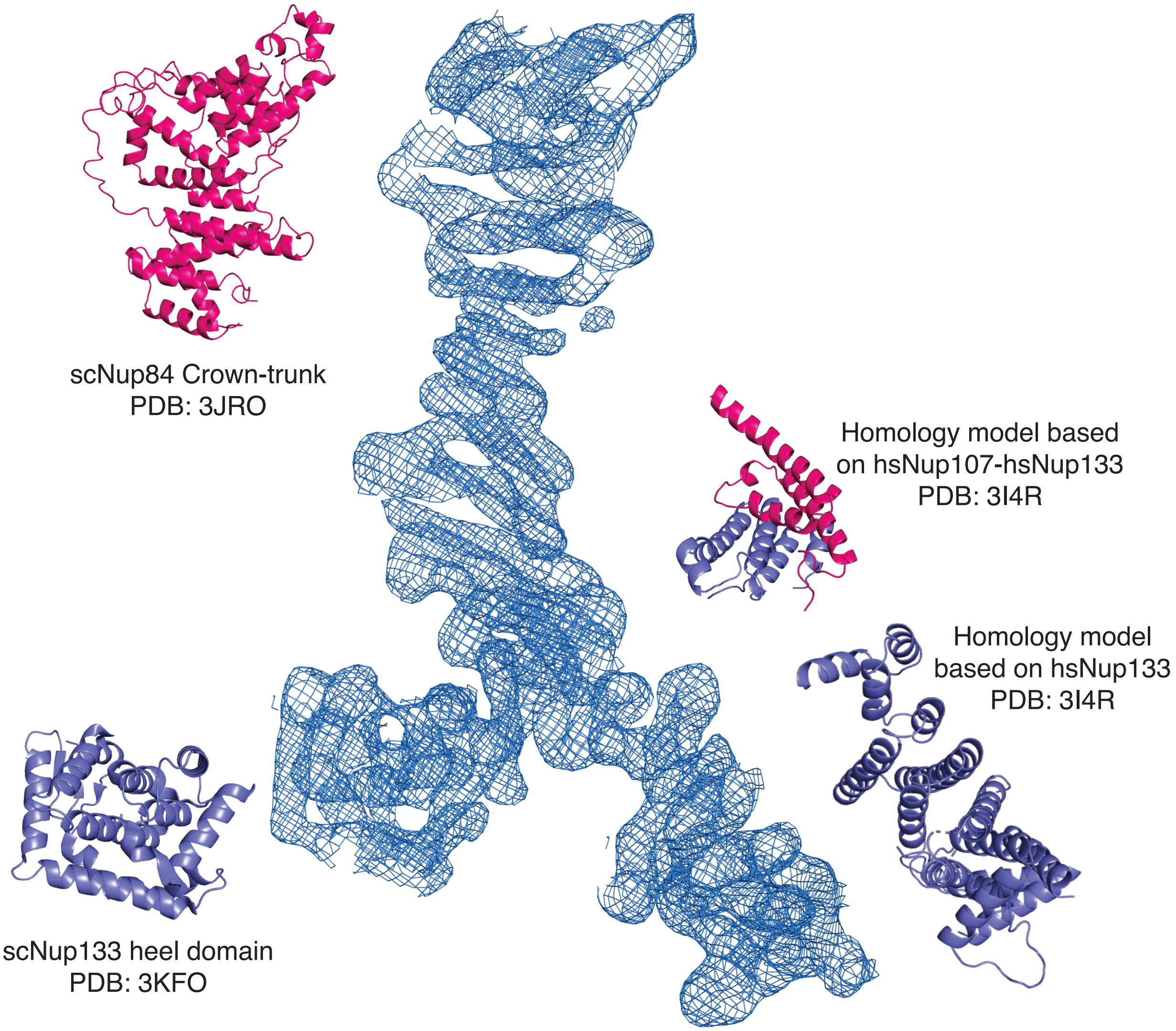

Supplementary Figure 1.

Electron density map for the Nup84-Nup133$_{CTD}$ structure.

Final $2F_O$-F_C electron density map contoured at 1.5 σ with fragments used to build the structure. Density for VHH-SAN8 was excluded for clarity.

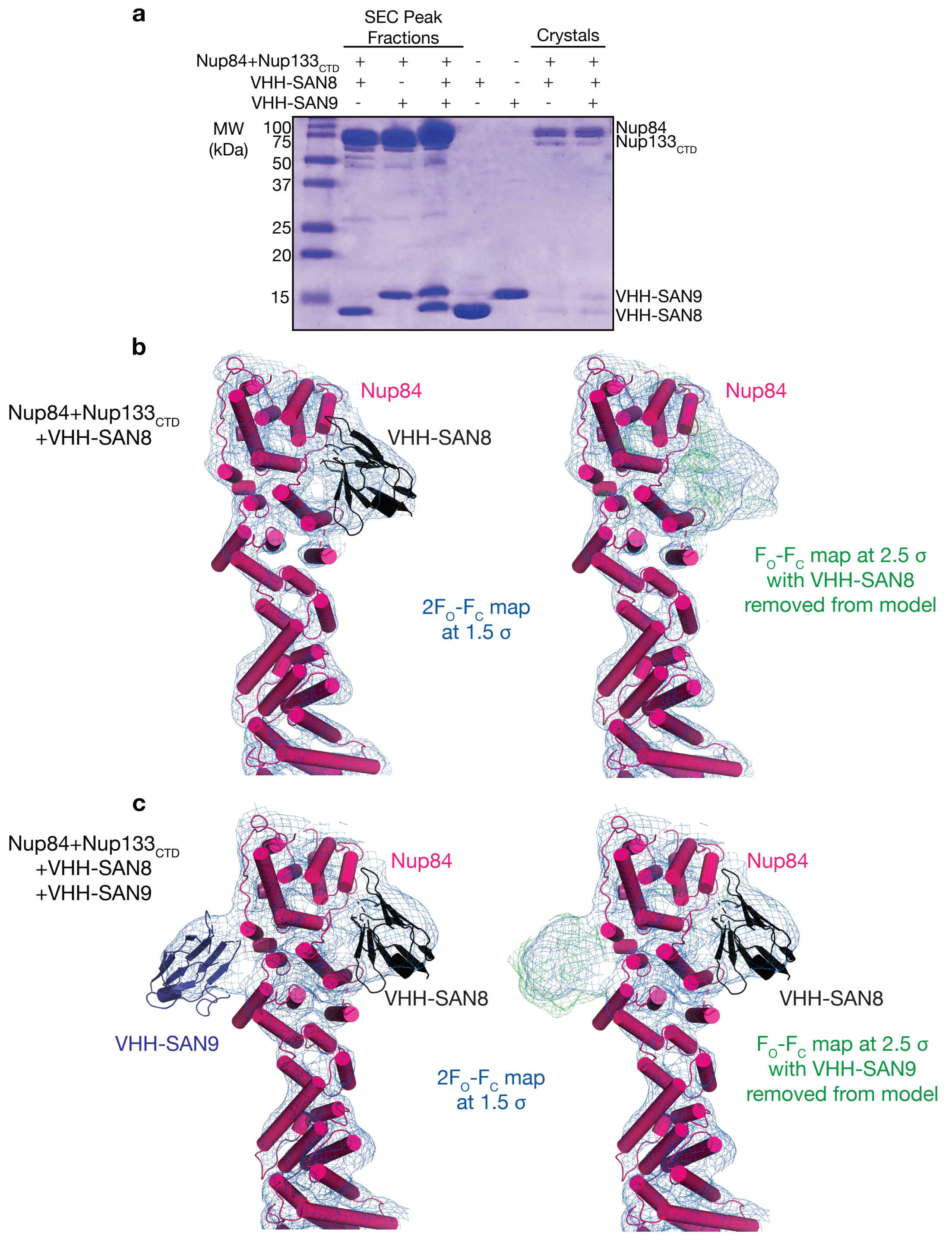

Supplementary Figure 2.

VHH-SAN8 and VHH-SAN9 are present in the structures.

(a) SDS-PAGE gel of purified Nup84-Nup133$_{CTD}$-VHH complexes and crystals of Nup84-Nup133$_{CTD}$-VHH-SAN8 and Nup84-Nup133$_{CTD}$-VHH-SAN8/9 complexes. The gel is representative of two independent experiments. (b) Binding location of VHH-SAN8 shown by density in both 2F_O-F_C (left) and F_O-F_C omit map when VHH-SAN8 is removed from the structure (right). (c) Same analysis as in (b) with the structure of Nup84-Nup133$_{CTD}$-VHH-SAN8/9.

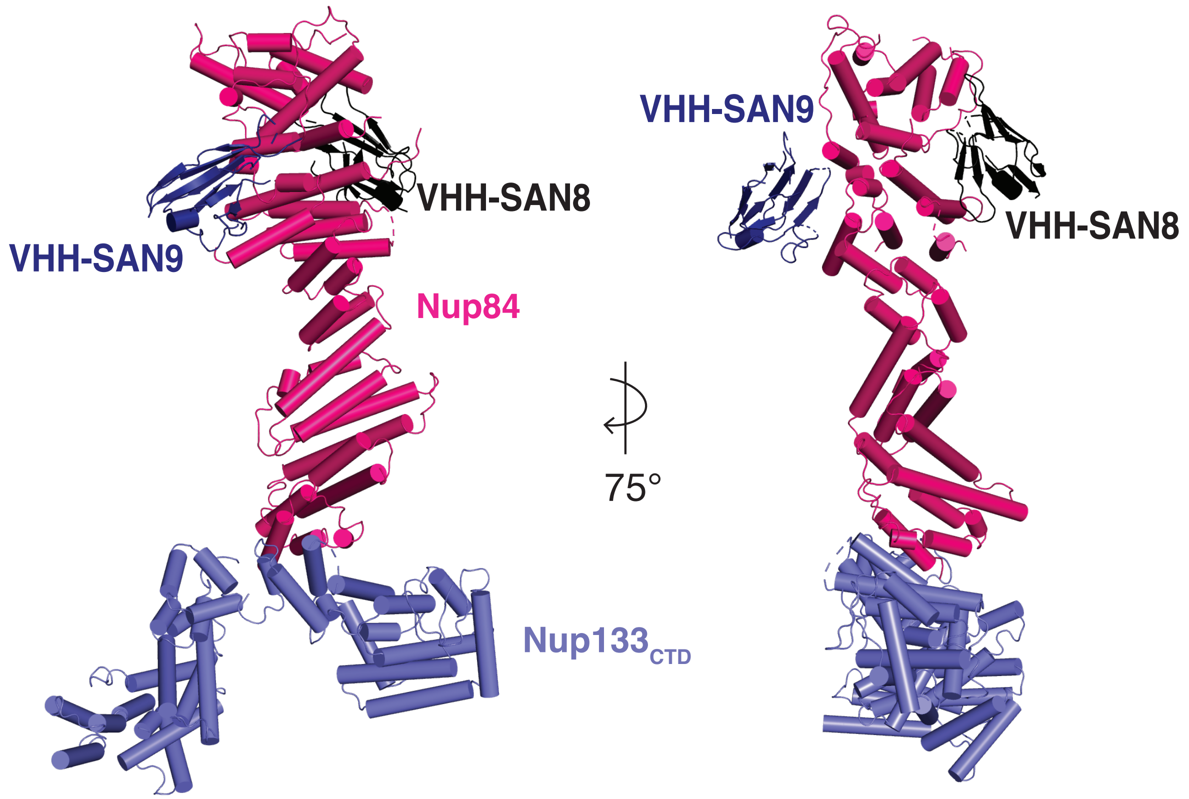

Supplementary Figure 3.

Structure of the *S. cerevisiae* Nup84-Nup133$_{CTD}$-VHH-SAN8/9 complex.

Structure of Nup84-Nup133$_{CTD}$-VHH-SAN8, with Nup84 in pink, Nup133$_{CTD}$ in purple, and VHH-SAN8 in black, VHH-SAN9 shown in navy.

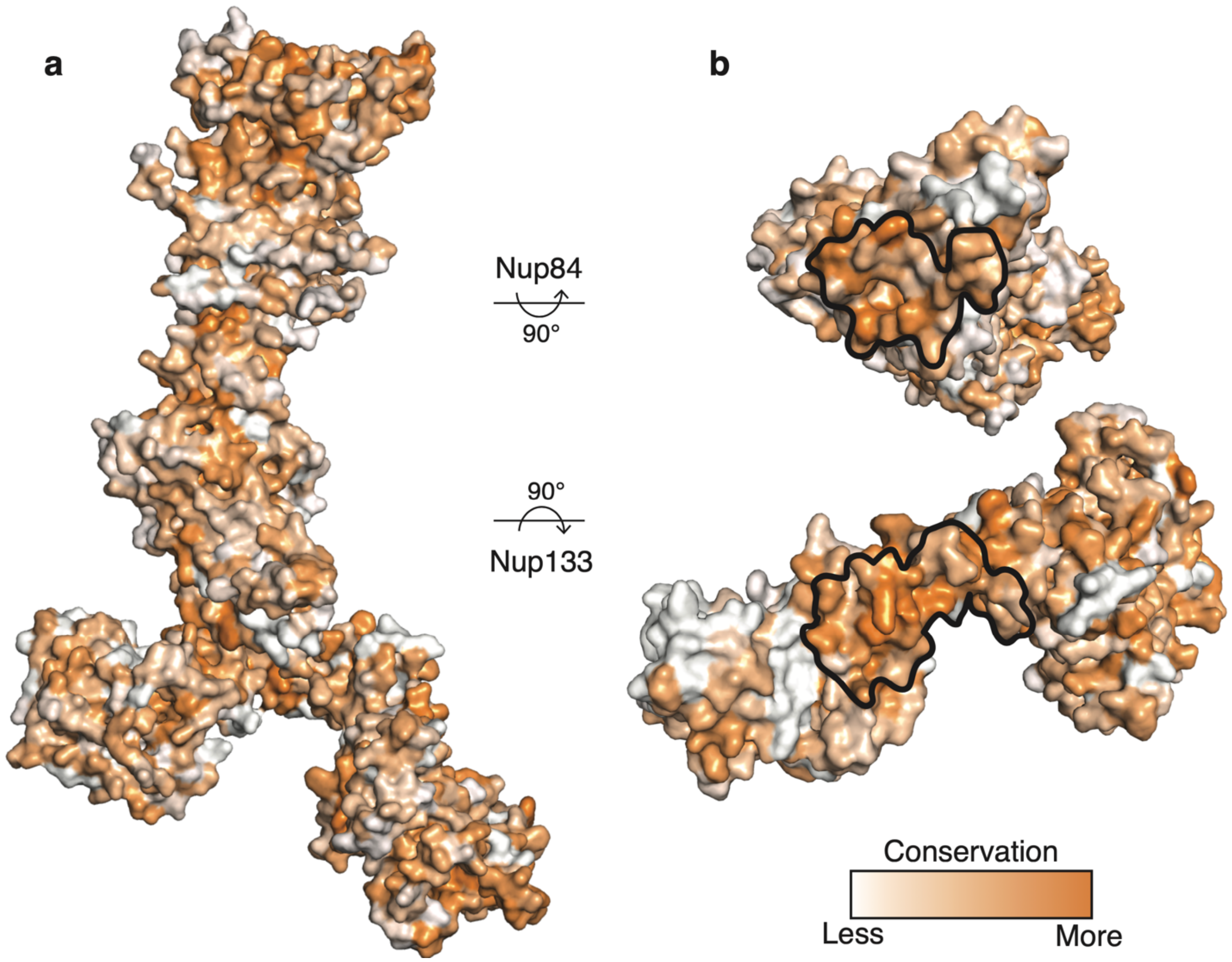

Supplementary Figure 4.

Conservation of Nup84-Nup133$_{CTD}$.

(a) Surface rendering of Nup84-Nup133$_{CTD}$ with a color gradient depicting conservation across diverse eukaryotes. (b) Open-book depiction of the interface between Nup84 and Nup133. The binding interface is outlined in black.

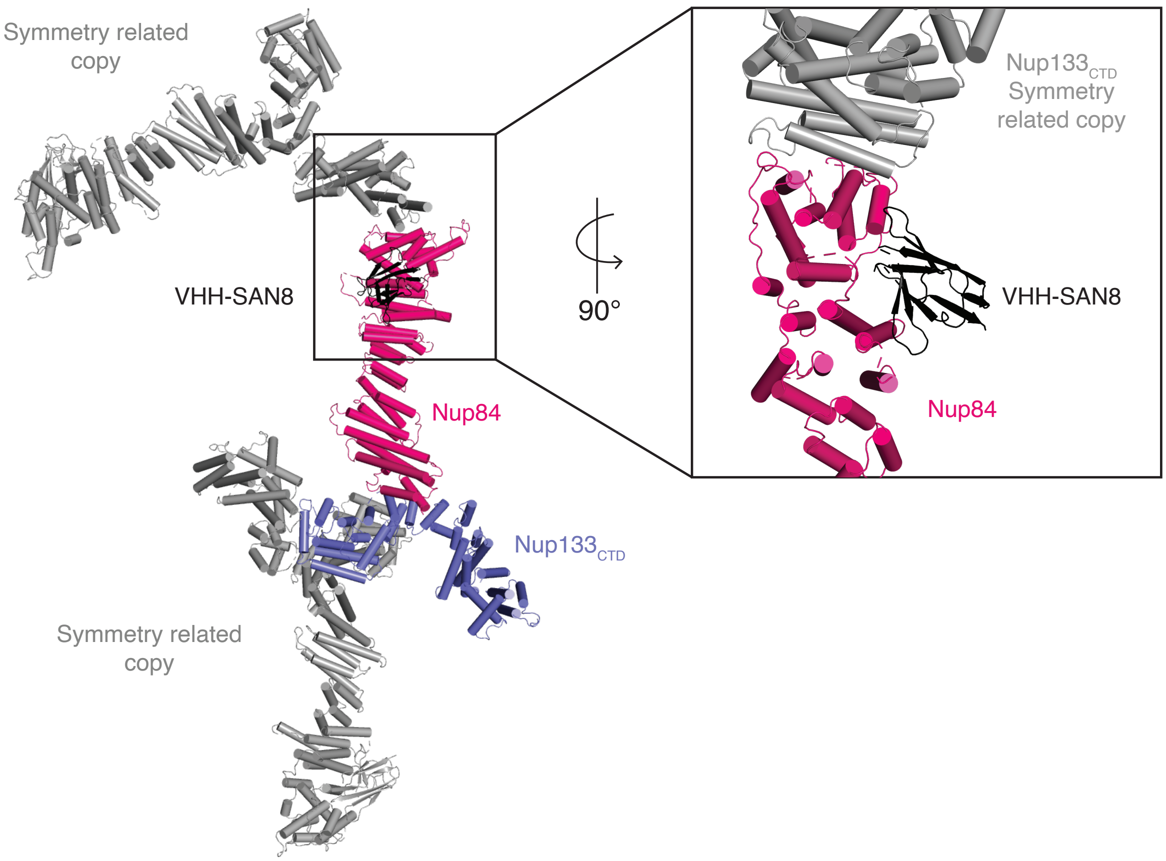

Supplementary Figure 5.

VHH-SAN8 is critical for rigidifying a crystal packing interface.

Depiction of packing interactions within the crystal. Two symmetry related copies are shown for Nup84-Nup133$_{CTD}$-VHH-SAN8 (gray). Inset shows a closer view of the interaction between Nup84 and a symmetry related copy of Nup133$_{CTD}$.

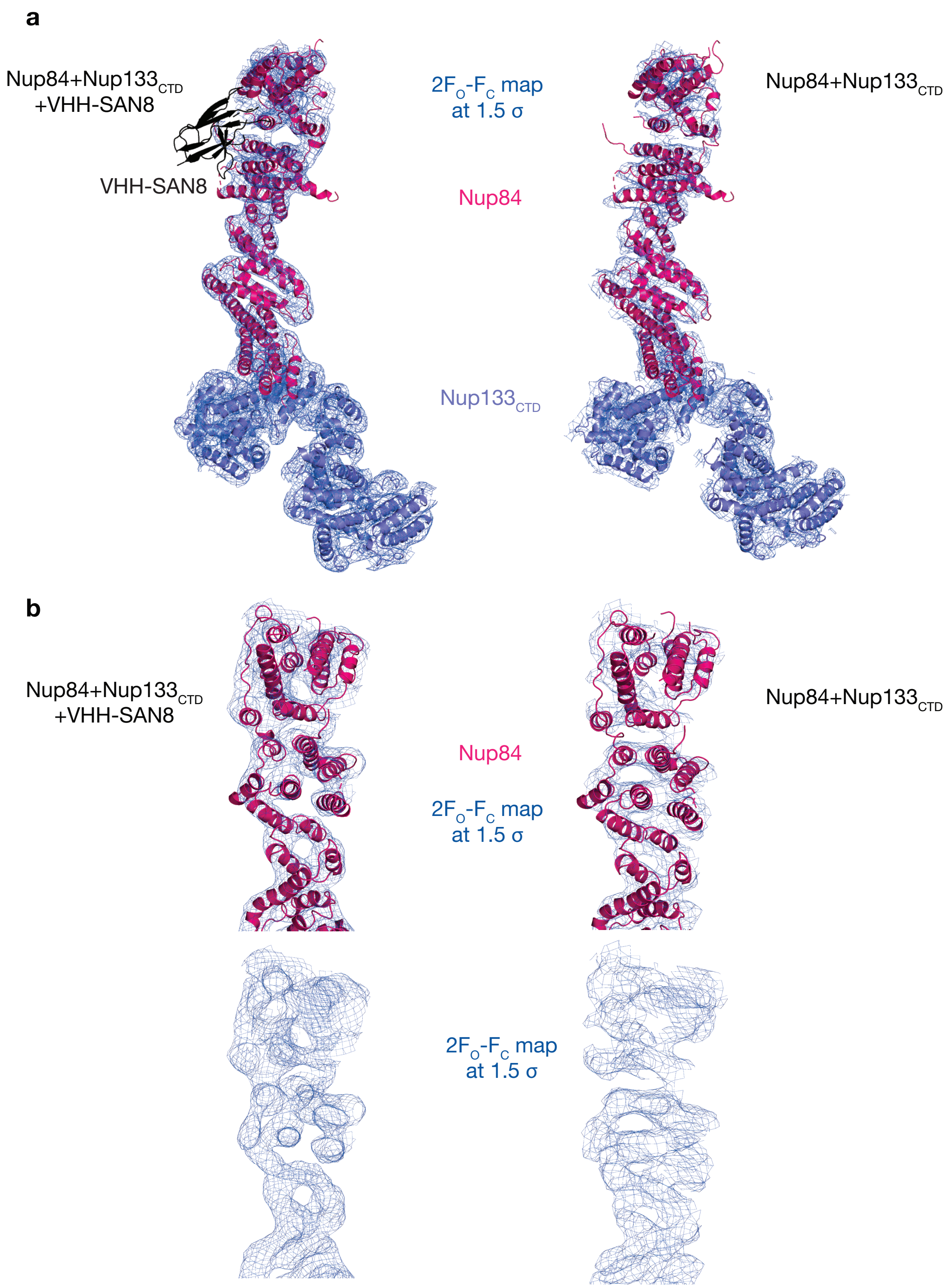

Supplementary Figure 6.

VHH-SAN8 rigidifies Nup84 crown within the crystal.

(a) Comparison of the $2F_O$-F_C maps for the crystal structures of Nup84-Nup133$_{CTD}$-VHH-SAN8 (left) and apo-Nup84-Nup133$_{CTD}$ (right). Map density contoured at 1.5σ. (b) Comparison as in (a), with emphasize on the Nup84$_{crown\text{-}trunk}$ area, with the model of Nup84 positioned (above) and without (below). While both unit cells are nearly the same, the nanobody-bound structure is much better defined and has

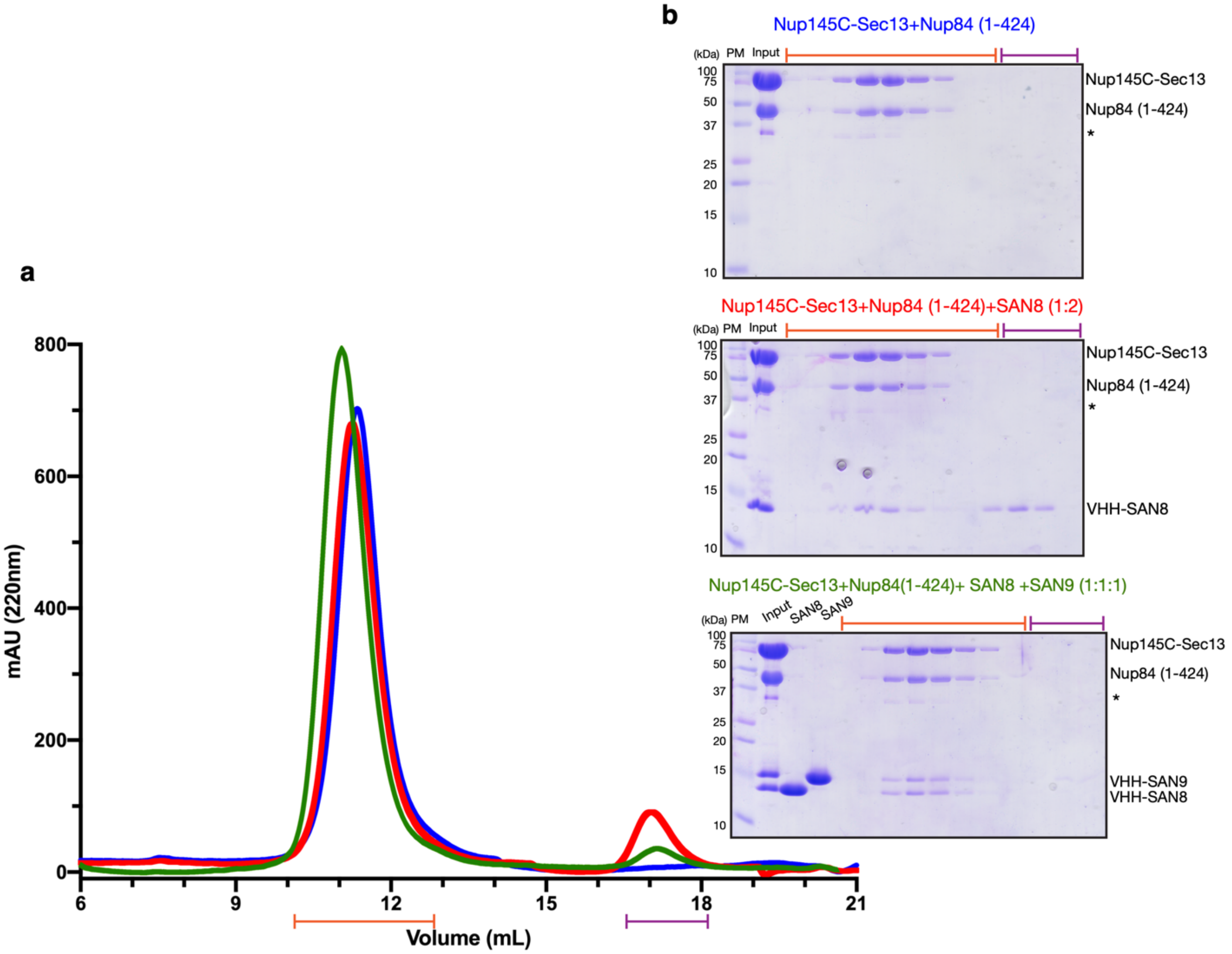

Supplementary Figure 7.

VHH-SAN8, VHH-SAN9 bind Nup84 with in complex with Nup145C.

(a) Size exclusion chromatography (SEC) for Nup145C-Sec13$_{fusion}$+Nup84$_{1-424}$ (blue), pre-incubated with VHH-SAN8 (1:2 molar ratio) (red), or pre-incubated with VHH-SAN8 and VHH-SAN9 (1:1:1 molar ratio) (green). (b) SDS-PAGE analysis of the SEC fractions indicated for each SEC experiment. *indicates contaminant protein. Co-elution of VHH-SAN8 and VHH-SAN8/9 shows that both nanobodies can bind Nup84 while in complex with Nup145C. Each panel is representative of two independent experiments.

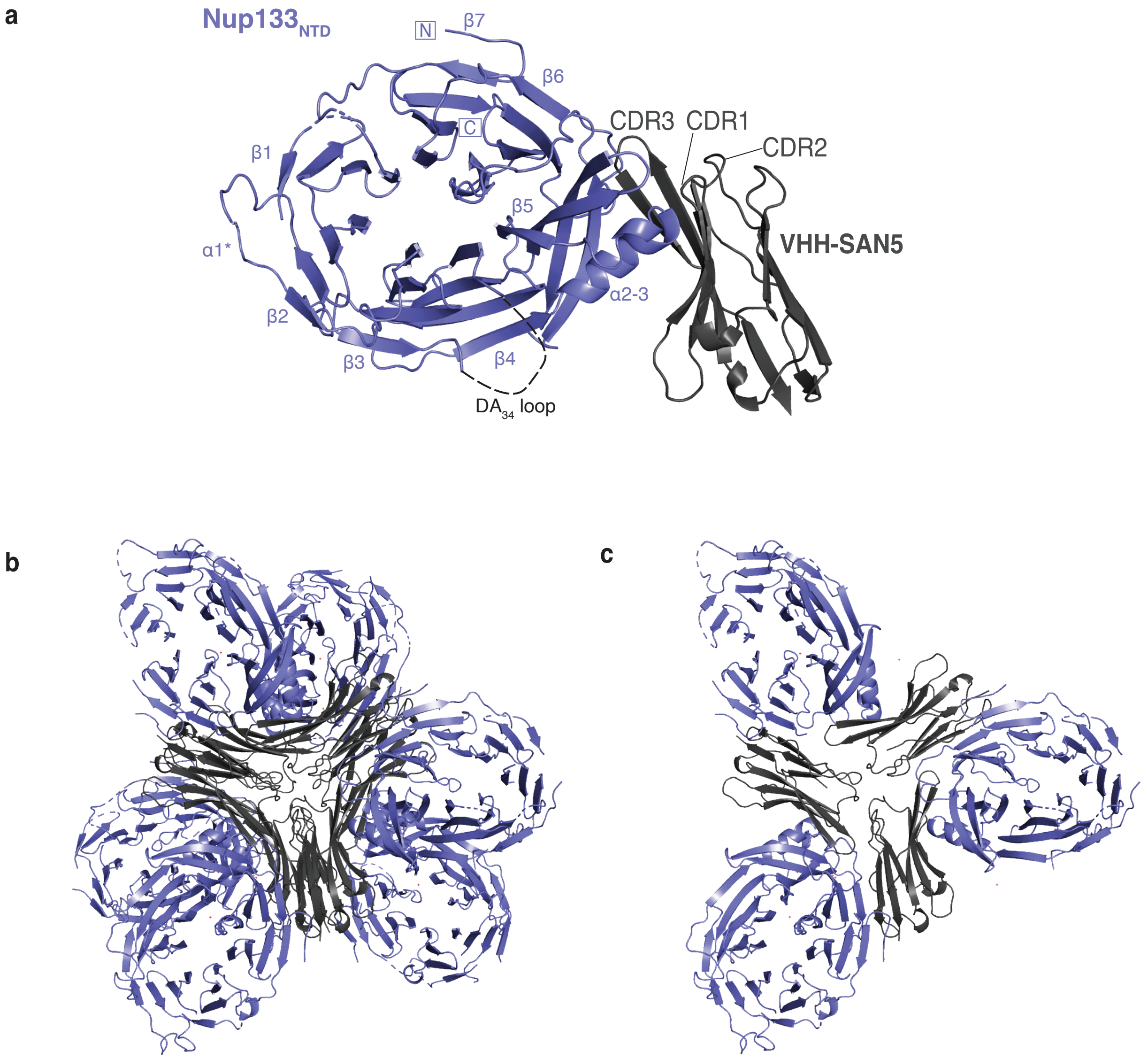

Supplementary Figure 8.

Structure of Nup133$_{NTD}$-VHH-SAN5 complex.

(a) Nup133$_{NTD}$-VHH-SAN5, with Nup133$_{NTD}$ in purple and VHH-SAN5 in dark gray. N and C termini are indicated, and β-sheets and α-helices are labeled on Nup133$_{NTD}$. Complementarity determining region (CDR) loops are labeled on VHH-SAN5. (b) Packing of Nup133$_{NTD}$-VHH-SAN5 in the asymmetric unit. Colors as in (a). (c) Heterohexameric assembly within the asymmetric unit highlighting packing interfaces mediated by VHH-SAN5.

Supplementary Table 1. BackPhyre Structural Homology Results

Table includes hits that cover >100 amino acids and >30% confidence.

Protein	Description	Alignment coverage (%)	Confidence	% Sequence ID
Nic96	Scaffold nucleoporin, Nic96/inner ring complex	224-637 (63)	98.7	18
Nup145C	Scaffold nucleoporin, Y complex	1,027-1,284 (39)	97.2	16
Sec31	COPII vesicle coat component	643-875 (35)	95.1	18
Sea4	SEA complex, lysosomal membrane interaction and autophagy	772-996 (34)	79.0	25
Sec16	COPII vesicle coat assembly	255-340 (13)	72.5	17
Nup85	Scaffold nucleoporin, Y complex	465-566 (15)	31.5	14

Supplementary Table 2. List of Primer Sequences for Cloning

Name	Forward primer (5'-3')	Reverse primer (5'-3')	Purpose
Nup133 NTD insert	GTTCTGTTCCAGGGGCCCGGATTCGATAATTCCAAGGTTTTCACAG	CCAGACTCGAGGGTACCTTACTACTCTTTCACAAAAAACTGTAGCACC	Amplify Nup133 NTD for Gibson assembly
pET28 vector open for Nup133 NTD	GGTGCTACAGTTTTTTGTGAAAGAGTAGTAAGGTACCCTCGAGTCTGG	CTGTGAAAACCTTGGAATTATCGAATCCGGGCCCCTGGAACAGAAC	Amplify pET28 6His vector for Gibson assembly
Nup133 ALPS mutant	GGAGGTGGGGGATCCCGAAATGGACCTATCCTCGG	GGATCCCCCACCTCCAGGTTTATTTAATAGTTTGCCTAACTTCAG	Inverse PCR primers to make Nup133 ALPS mutant
Nanobody hit insert	GACTGGTGGCTCAAGTGGATCCATGCAGGTGCAGCTCGTG	ATGCGGCCGTGTACATTATCACTATTATGCGGCACGCGGTTCC	Amplify nanobody hit sequences for Gibson assembly
Open pAH His14bdSUMO	GGAACCGCGTGCCGCATAATAGTGATA	GCATGGATCCACTTGAGCCACCAGTC	Amplify pAH His14BdSUMO vector for nanobody expression Gibson assembly
