# Supplementary material for: Yeast Nup84-Nup133 complex structure details flexibility and reveals conservation of the membrane anchoring ALPS motif
Source: Nat Commun. 2020 Nov 27;11:6060. doi: 10.1038/s41467-020-19885-5 (PMC7695694; doi:10.1038/s41467-020-19885-5)
Supplement: Supplementary file 3 — Reporting Summary [file 41467_2020_19885_MOESM3_ESM.pdf]

nature research

Corresponding author(s): Thomas U. Schwartz

Last updated by author(s): 10/22/2020

Reporting Summary

Nature Research wishes to improve the reproducibility of the work that we publish. This form provides structure for consistency and transparency in reporting. For further information on Nature Research policies, see our Editorial Policies and the Editorial Policy Checklist.

Statistics

For all statistical analyses, confirm that the following items are present in the figure legend, table legend, main text, or Methods section.

n/a	Confirmed	
☐	☒	The exact sample size (*n*) for each experimental group/condition, given as a discrete number and unit of measurement
☐	☒	A statement on whether measurements were taken from distinct samples or whether the same sample was measured repeatedly
☒	☐	The statistical test(s) used AND whether they are one- or two-sided<br>*Only common tests should be described solely by name; describe more complex techniques in the Methods section.*
☒	☐	A description of all covariates tested
☒	☐	A description of any assumptions or corrections, such as tests of normality and adjustment for multiple comparisons
☒	☐	A full description of the statistical parameters including central tendency (e.g. means) or other basic estimates (e.g. regression coefficient) AND variation (e.g. standard deviation) or associated estimates of uncertainty (e.g. confidence intervals)
☒	☐	For null hypothesis testing, the test statistic (e.g. *F*, *t*, *r*) with confidence intervals, effect sizes, degrees of freedom and *P* value noted<br>*Give P values as exact values whenever suitable.*
☒	☐	For Bayesian analysis, information on the choice of priors and Markov chain Monte Carlo settings
☒	☐	For hierarchical and complex designs, identification of the appropriate level for tests and full reporting of outcomes
☒	☐	Estimates of effect sizes (e.g. Cohen's *d*, Pearson's *r*), indicating how they were calculated

Our web collection on statistics for biologists contains articles on many of the points above.

Software and code

Policy information about availability of computer code

Data collection	X-ray diffraction data was collected at the Advanced Photon Source NE-CAT beamline 24-ID-C.
Data analysis	HKL-2000 v720, Phenix v1.18.2, Coot 0.9, PyMOL v2.3.4 UCSF, Chimera v1.14

For manuscripts utilizing custom algorithms or software that are central to the research but not yet described in published literature, software must be made available to editors and reviewers. We strongly encourage code deposition in a community repository (e.g. GitHub). See the Nature Research guidelines for submitting code & software for further information.

Data

Policy information about availability of data

All manuscripts must include a data availability statement. This statement should provide the following information, where applicable:

- Accession codes, unique identifiers, or web links for publicly available datasets
- A list of figures that have associated raw data
- A description of any restrictions on data availability

Coordinates and structure factors have been deposited to the Protein Data Bank with accession codes 6X02, 6X03, 6X04, and 6X05.

Field-specific reporting

Life sciences study design

All studies must disclose on these points even when the disclosure is negative.

Sample size	X-ray diffraction data was collected from a single crystal, but many crystals were grown, and several datasets were collected, that primarily differed in resolution, but not in spacegroup or unit cell dimensions. For the yeast experiments in Figure 6b,c, at least 50 cells were examined.
Data exclusions	No data were excluded from the analyses.
Replication	Crystallization conditions were repeat at least 20 times. All biochemical data was acquired at least in triplicates.
Randomization	Not Applicable. Randomization is not possible in X-ray crystallographic studies as it is essential to have the full information about the material used in the study in order to determine 3-dimensional structures.
Blinding	Blinding is not possible in X-ray crystallographic studies as it is essential to have full information about the material in order to determine 3-dimensional structures.

Reporting for specific materials, systems and methods

We require information from authors about some types of materials, experimental systems and methods used in many studies. Here, indicate whether each material, system or method listed is relevant to your study. If you are not sure if a list item applies to your research, read the appropriate section before selecting a response.

Materials & experimental systems

n/a	Involved in the study	
☐	☒	Antibodies
☒	☐	Eukaryotic cell lines
☒	☐	Palaeontology and archaeology
☒	☐	Animals and other organisms
☒	☐	Human research participants
☒	☐	Clinical data
☒	☐	Dual use research of concern

Methods

n/a	Involved in the study	
☒	☐	ChIP-seq
☒	☐	Flow cytometry
☒	☐	MRI-based neuroimaging

Antibodies

Antibodies used	Nanobodies were generated as part of this study and their characteristics are described.
Validation	All validation data (in vitro binding data, structural data) are described in the manuscript.
